# Supplementary material for: Nutraceutical Potential of Djulis (Chenopodium formosanum) Hull: Phytochemicals, Antioxidant Activity, and Liver Protection
Source: Antioxidants (Basel). 2024 Jun 13;13(6):721. doi: 10.3390/antiox13060721 (PMC11201270; doi:10.3390/antiox13060721)
Supplement: Supplementary file 1 [file antioxidants-13-00721-s001.zip › antioxidants-3012203-supplementary.pdf]

Supplementary Table 1. Ranking 1 to 20 of the composition of three strains of Djulis hull and kernel crude extracts

Rank	Compound name	Parent mass	Spectra number of hull	Spectra number of kernel
1	1-Hexadecanoyl-2-octadecadienoyl-*sn*-glycero-3-phosphocholine	758.569 (M+H)	115	327
2	1-Hexadecanoyl-2-(9*Z*-octadecenoyl)-*sn*-glycero-3-phosphocholine	782.569 (M+Na)	148	283
3	AEG(o-16:2/18:2)	575.503 (M+H)	177	195
4	Arachidonoylthio-PC	784.584 (M+H)	22	140
5	2-Linoleoyl-1-palmitoyl-*sn*-glycero-3-phosphoethanolamine	716.522 (M+H)	6	120
6	TAG (16:0/18:1/18:2)	874.785 ($M+NH_4$)	110	0
7	Trimethyl(2-{[2-[octadec-11-enoyloxy]-3-[octadec-9-enoyloxy]propyl phosphonato]oxy}ethyl)azanium	786.595 (M+H)	19	82
8	PC(18:1/18:1)	785.585 (M+H)	12	81
9	PC(16:0/18:0)	761.583 (M+H)	0	80
10	Linoleic acid ethyl ester	309.278 (M+H)	2	71
11	AEG(o-18:4/16:1)	573.487 (M+H)	44	25
12	Rutin	611.160 ($M+H^+$)	36	26
13	3-[Octadec-11-enoyloxy]-2-[octadec-9-enoyloxy]propyl octadeca-9.12-dienoate	900.801 ($M+NH_4$)	62	0
14	Monolinolenin (9c,12c,15c)	353.268 (M+H)	36	15
15	LPC 18:2	520.340 (M+H)	22	28
16	Lyso-PC(16:0)	496.340 (M+H)	19	22
17	1-Myristoyl-2-stearoyl-*sn*-glycero-3-phosphocholine	734.569 (M+H)	0	40
18	Kaempferol-3-*O*-rutinoside	595.166 (M+H)	26	12
19	1-(9Z-Octadecenoyl)-*sn*-glycero-3-phosphocholine	522.355 (M+H)	11	22
20	Flavovilloside	757.218 (M+H)	13	19

Supplementary Table 2. Ranking 1 to 15 in the composition of flavonoid cluster of Djulis hull and kernel crude extracts

Rank	Compound name	Parent mass	Spectra number of hull	Spectra number of kernel
1	Rutin	611.160 (M+H$^+$)	36	26
2	Kaempferol-3-*O*-rutinoside	595.166 (M+H)	26	12
3	Flavovilloside	757.218 (M+H)	13	19
4	Quercetin 3-(2*R*-apiosylrutinoside)	743.202 (M+H)	6	13
5	Quercitrin	449.107 (M+H$^+$)	15	4
6	Hyperoside	465.102 (M+H$^+$)	8	9
7	Kaempferol-3-rhamninoside	741.223 (M+H)	7	7
8	Isorhamnetin-3-*O*-rutinoside	625.176 (M+H)	3	11
9	NCGC00179918-02	565.155 (M+H)	8	3
10	Kaempferol-3-*O*-rhamnoside-7-*O*-rhamnoside	579.170 (M+H)	8	2
11	Kaempferol-3-*O*-pentoxyl-pentoside	551.139 (M+H)	3	1
12	NCGC00380680-01	727.208 (M+H$^+$)	0	4
13	Quercetin 3-xylosyl-(1→2)-alpha-L-arabinofuranoside	567.134 (M+H)	3	0
14	NCGC00385381-01	597.145 (M+H$^+$)	0	2
15	Xanthorhamnin	771.234 (M+H$^+$)	0	2

NCGC00179918-02: 5-hydroxy-2-(4-hydroxyphenyl)-7-(3,4,5-trihydroxy-6-methyloxan-2-yl)oxy-3-(3,4,5-trihydroxyoxan-2-yl)oxychromen-4-one.

NCGC00380680-01: 5-hydroxy-3-[(2*S*,3*R*,4*R*,5*S*)-3-hydroxy-5-(hydroxymethyl)-4-[(2*S*,3*R*,4*S*,5*S*,6*R*)-3,4,5-trihydroxy-6-(hydroxymethyl)oxan-2-yl]oxyoxolan-2-yl]oxy-2-(4-hydroxyphenyl)-7-[(2*S*,3*R*,4*R*,5*R*,6*S*)-3,4,5-trihydroxy-6-methyloxan-2-yl]oxychromen-4-one.

NCGC00385381-01: 2-(3,4-dihydroxyphenyl)-5,7-dihydroxy-3-[(2*S*,3*R*,4*S*,5*S*,6*R*)-3,4,5-trihydroxy-6-[[(2*S*,3*R*,4*S*,5*R*)-3,4,5-trihydroxyoxan-2-yl]oxymethyl]oxan-2-yl]oxychromen-4-one.

Supplementary Table 3. Ranking 1 to 12 in the composition of the flavonoid cluster of three strains of Djulis hull crude extract

Rank	Compound name	Parent mass	Spectra number of red hull	Spectra number of orange hull	Spectra number of yellow hull
1	Rutin	611.160 $(M+H^{+})$	12	13	11
2	Kaempferol-3-*O*-rutinoside	595.166 (M+H)	8	9	9
3	Quercitrin	449.107 $(M+H^{+})$	6	5	4
4	Flavovilloside	757.218 (M+H)	3	5	5
5	Hyperoside	465.102 $(M+H^{+})$	2	3	3
6	NCGC00179918-02	565.155 (M+H)	2	3	3
7	Kaempferol-3-*O*-rhamnoside-7-*O*-rhamnoside	579.170 (M+H)	2	4	2
8	Kaempferol-3-rhamninoside	741.223 (M+H)	2	3	2
9	Quercetin 3-(2*R*-apiosylrutinoside)	743.202 (M+H)	2	2	2
10	Isorhamnetin-3-*O*-rutinoside	625.176 (M+H)	1	1	1
11	Kaempferol-3-*O*-pentoxyl-pentoside	551.139 (M+H)	1	1	1
12	Quercetin 3-xylosyl-(1→2)-alpha-L-arabinofuranoside	567.134 (M+H)	1	1	1

NCGC00179918-02: 5-hydroxy-2-(4-hydroxyphenyl)-7-(3,4,5-trihydroxy-6-methyloxan-2-yl)oxy-3-(3,4,5-trihydroxyoxan-2-yl)oxychromen-4-one.
